# Tantalum-Doped TiO_2_ Prepared by Atomic Layer Deposition and Its Application in Perovskite Solar Cells

**DOI:** 10.3390/nano11061504

**Published:** 2021-06-07

**Authors:** Chia-Hsun Hsu, Ka-Te Chen, Ling-Yan Lin, Wan-Yu Wu, Lu-Sheng Liang, Peng Gao, Yu Qiu, Xiao-Ying Zhang, Pao-Hsun Huang, Shui-Yang Lien, Wen-Zhang Zhu

**Affiliations:** 1 School of Opto-Electronic and Communication Engineering, Xiamen University of Technology, Xiamen 361024, China; chhsu@xmut.edu.cn (C.-H.H.); 1922031007@stu.xmut.edu.cn (K.-T.C.); xyzhang@xmut.edu.cn (X.-Y.Z.); wzzhu@xmut.edu.cn (W.-Z.Z.); 2 Key Laboratory of Green Perovskites Application of Fujian Province Universities, Fujian Jiangxia University, Fuzhou 350108, China; lingyanlin@fjjxu.edu.cn (L.-Y.L.); yuqiu@fjjxu.edu.cn (Y.Q.); 3 College of Electronics and Information Science, Fujian Jiangxia University, Fuzhou 350108, China; 4 Department of Materials Science and Engineering, Da-Yeh University, Changhua 51591, Taiwan; wywu@mail.dyu.edu.tw; 5 CAS Key Laboratory of Design and Assembly of Functional Nanostructures, Fujian Provincial Key Laboratory of Nanomaterials, Fujian Institute of Research on the Structure of Matter, Chinese Academy of Sciences, Fuzhou 350002, China; lushengliang@fjirsm.ac.cn (L.-S.L.); peng.gao@fjirsm.ac.cn (P.G.); 6 School of Information Engineering, Jimei University, Xiamen 361021, China; ph.huang@jmu.edu.cn; 7 Fujian Key Laboratory of Optoelectronic Technology and Devices, Xiamen University of Technology, Xiamen 361024, China

**Keywords:** tantalum, titanium oxide, atomic layer deposition, bubbler temperature, perovskite solar cell, electron transport layer

## Abstract

Tantalum (Ta)-doped titanium oxide (TiO_2_) thin films are grown by plasma enhanced atomic layer deposition (PEALD), and used as both an electron transport layer and hole blocking compact layer of perovskite solar cells. The metal precursors of tantalum ethoxide and titanium isopropoxide are simultaneously injected into the deposition chamber. The Ta content is controlled by the temperature of the metal precursors. The experimental results show that the Ta incorporation introduces oxygen vacancies defects, accompanied by the reduced crystallinity and optical band gap. The PEALD Ta-doped films show a resistivity three orders of magnitude lower than undoped TiO_2_, even at a low Ta content (0.8–0.95 at.%). The ultraviolet photoelectron spectroscopy spectra reveal that Ta incorporation leads to a down shift of valance band and conduction positions, and this is helpful for the applications involving band alignment engineering. Finally, the perovskite solar cell with Ta-doped TiO_2_ electron transport layer demonstrates significantly improved fill factor and conversion efficiency as compared to that with the undoped TiO_2_ layer.

## 1. Introduction

Titanium dioxide (TiO_2_) thin films are widely studied owing to their optoelectrical properties that fulfill the requirements of numerous applications such as dye-sensitized photovoltaic devices [1], photocatalysis [2], and perovskite solar cells (PSCs) [3]. In order to enhance properties, the TiO_2_ films are commonly doped with transition metal, rare metal, or noble metal ions [4,5,6,7,8,9]. Some studies indicate that the electron-hole recombination could be reduced due to the generation of the charge trapping centers by foreign ions [10]. A moderate amount of metal dopants promotes the excitons separation and thus improves the mobility of photogenerated carriers [11,12,13]. Due to the wide forbidden energy gap and the absorption only at UV-light region, the TiO_2_ films are intentionally doped in order to reduce the energy gap and extend the light absorption to visible light in some applications [14]. The reduced band gap results from the generation of oxygen vacancies produced simultaneously through doping, introducing shallow energy levels below the conduction band edge [15,16]. Zhao et al. reported that doped TiO_2_ with suitable metal ions can effectively improve the electron transport of TiO_2_ [17]. Among various dopants, Ta^5+^ has an ionic radius (0.64 Å) very close to Ti^4+^ (0.61 Å), and thus it is able to be incorporated into the TiO_2_ lattice without severe strain or secondary phase production [18]. Doping with Ta is also more effective than with niobium, and has lower formation energy consumption and superior thermodynamic stability compared to doping with nitrogen [13]. Ta-doped TiO_2_ thin films can be prepared by various methods such as sputtering [19], sol-gel spin-coating [13], and chemical vapor deposition [20]. Recently, due to the demands of slim and thin electronics or the requirements of depositing films on high aspect ratio substrates, atomic layer deposition (ALD) technique receives great attention because of its pinhole-free deposition process, accurate thickness control at sub-nanometer level, and high conformality [21]. The Ta incorporation into ALD TiO_2_ films is mostly carried out by using the Ta_2_O_5_/TiO_2_ multilayer structure, and controlled by the ALD cycle ratio of the two metal oxides [18,22].

As for planar perovskite solar cells, the electron transport layer (ETL) plays a crucial role in electron transport and hole blocking. Due to the fact that ETLs are mostly prepared using the sol-gel process, the layer may contain pinholes or have low density, rendering holes possible to pass the ETL to increase the recombination rate. A compact layer is usually inserted at the sol-gel ETL/transparent electrode interface to improve the hole blocking ability. Thanks to the pinhole-free structure and highly conformal coverage, a thin ALD layer is able to be used as both ETL and compact layer for the perovskite solar cells [23]. However, the conduction band edge of TiO_2_ (about −3.9 eV) is shallower than that of cesium formamidinium-based Cs_x_FA_1–x_Pb(I_1–y_Br_y_)_3_ (about −4.2 eV), which is one of the most promising classes of perovskite light absorber for solar cells due to its wide band gap and higher durability compared to MAPbI_3_ [24]. The conduction band mismatch at the TiO_2_ ETL/perovskite interface causes a barrier that is theoretically unfavorable for electron transport. Development of doped TiO_2_ ETLs is one possible way to have a better band alignment and an improved PSC performance.

In the present study, Ta-doped TiO_2_ films are grown by using plasma enhanced ALD (PEALD), and used as an ETL for PSCs. The metal precursors tantalum ethoxide (Ta(OEt)_5_) as Ta dopant source and titanium isopropoxide (TTIP) as Ti source are simultaneously fed into the deposition chamber. The precursor output flows are controlled by the bubbler temperature, which is varied from 70–90 °C to obtain different Ta doping ratio. The effects of the bubbler temperature on the structural, electrical, and optical properties of the Ta-doped TiO_2_ films are investigated. The adjustments of the band gap, conduction band, and valence band positions are presented. Finally, the Ta-doped TiO_2_ ETL is applied to PSC fabrication, and the improved conversion efficiency and hysteresis is demonstrated and discussed.

## 2. Materials and Methods

### 2.1. Thin Film Preparation

Glass substrates with a size of 20 mm × 20 mm and a thickness of 1 mm were cleaned in an ultrasonic bath with deionized water, acetone, and ethanol for 15 min, and then dried in an oven at 70 °C for 30 min. The substrates were placed on the substrate holder in the deposition chamber of PEALD (R200 advance, Picosun Oy, Espoo, Finland). The used metal precursors were Ta(OEt)_5_ and TTIP (Aimou Yuan Scientific, Nanjing, China) as the Ta and Ti sources, respectively. Nitrogen with a purity of 99.999% was introduced to the precursor bubblers to transport the precursor vapors to the deposition chamber. The oxidant was provided by inductively coupled oxygen-argon mixed plasma generated by an RF power of 1500 W. The substrate temperature was 250 °C. The temperatures of the Ta(OEt)_5_ and TTIP bubblers were controlled from 70 to 90 °C by means of a heating jacket. The thickness of the Ta-doped TiO_2_ films was 60 nm. The undoped films were prepared under identical conditions, except for the use of the TTIP metal precursor alone. The detailed deposition parameters for the PEALD Ta-doped TiO_2_ films are summarized in Table 1.

### 2.2. Perovskite Solar Cell Fabrication

The 15 nm-thick ALD TiO_2_ films were deposited on the cleaned fluorine-doped tin oxide (FTO) substrates. Lead iodide, lead bromide, methylammonium bromide, and formamidinium iodide were dissolved with a molar ratio of 1:1.15:0.2:0.2 in *N*,*N*-dimethylformamide (DMF) and dimethyl sulfoxide (DMSO) with 4:1 volume ratio. The CsI dissolved in DMSO as 1.5 mol stock solution was further poured to give Cs_0.1_(FA_0.83_MA_0.17_)_0.9_Pb(I_0.83_Br_0.17_)_3_. The perovskite precursor solution was then spin-coated on ALD doped or undoped TiO_2_ ETL in two steps, the first spreading step with 1000 rpm for 10 s and the second step with 6000 rpm for 25 s. Chlorobenzene of 110 μL was sprayed on the spinning substrate at five seconds before the end of the second-step spin-coating process. Afterwards, an annealing process at 100 °C was performed for 60 min. Spiro-OMeTAD (Lumtec, New Taipei, Taiwan) of 50 uL was spin-coated on the perovskite with 4000 rpm for 30 s following cool down of the substrate back to room temperature. Finally, the cell fabrication was finished by evaporating gold films on the Spiro-OMeTAD. The devices had an active area of 0.1 cm^2^.

### 2.3. Characterization

The thickness of the films was determined using an ellipsometer (M-2000, J. A. Woollan Co., Lincoln, NE, USA). The ultraviolet-visible spectrometer (MFS630, Hong-Ming, New Taipei City, Taiwan) was employed to obtain the transmittance spectra. The X-ray diffraction apparatus (XRD, Rigaku TTRAXIII, Ibaraki, Japan) was used for characterizing the crystalline structure of the films. The X-ray photoelectron spectroscopy (XPS, ESCALAB 250Xi, Thermo Fisher, Waltham, MA, USA) was used in order to investigate the chemical states and elemental composition of the films. The Fermi-level and valance band position of the Ta-doped TiO_2_ films were obtained using UV photoelectron spectroscopy (UPS, ESCALAB Xi^+^, Thermo Fisher Scientific, Gloucester, UK) with He I source (photon energy of 21.2 eV). The resistivity of the films was examined by using a four-point probe (T2001A3, Ossila, Sheffield, UK). The current density–voltage (J–V) curves and solar cell external parameters such as open-circuit voltage (V_oc_), short-circuit current density (J_sc_), fill factor (FF), and conversion efficiency (η) were measured at AM1.5G (100 mW/cm^2^) using a solar simulator (Newport Oriel, Irvine, CA, USA).

## 3. Results and Discussion

**Figure 1:**
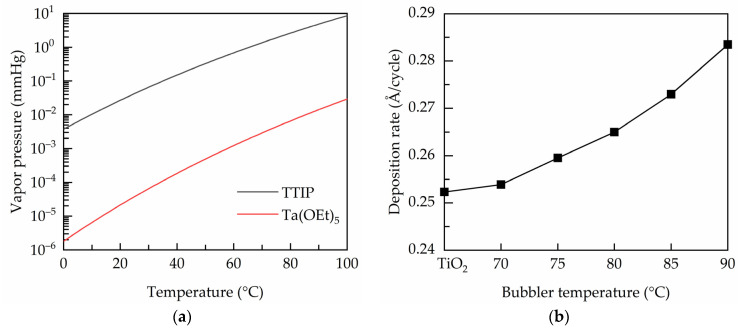
(**a**) Temperature-dependent vapor pressures of the TTIP and Ta(OEt)_5_ metal precursors. (**b**) Deposition rate of the PEALD films prepared at various bubbler temperatures.

In this work, Ta(OEt)_5_ precursor is used as the Ta dopant source, and co-injected with TTIP precursor into the deposition zone. The amount of the output precursor molecules is determined by the bubbler temperature controlling the vapor pressure of the metal precursors. Figure 1a shows the temperature-dependent vapor pressures of TTIP and Ta(OEt)_5_ as given by:

(1) $$P_{\mathrm{Ta}\left(\mathrm{OEt}\right)5}=10^{9.66-\frac{4288}{T}},$$

(2) $$P_{\mathrm{TTIP}}=10^{10.12-\frac{3425}{T}},$$

where *P* is the vapor pressure (unit in mmHg), and *T* is the bubbler temperature. Both of the metal vapor pressures increase with the bubbler temperature. It is believed that the film growth is related to the output vapors of metal precursors, which can be controlled by either varying the carrier gas flows or the bubbler temperature. The latter is adopted in this study. Figure 1b shows the deposition rates of the PEALD Ta-doped TiO_2_ films prepared at different bubbler temperatures. The value of the undoped film is also indicated for comparison. It is seen that the TiO_2_ has a deposition rate of 0.252 Å/cycle, which is similar to that reported by other groups [25,26]. The deposition rate is not much affected at the bubbler temperature of 70 °C, and then increases from 0.252 to 0.284 Å/cycle when the bubbler temperature increases from 70 to 90 °C. It is reported that the deposition rate of the ALD tantalum oxide is 0.5–1 Å/cycle [27,28], which is nearly two to four times higher than that of TiO_2_. This may be one explanation for the increased deposition rate for the Ta-doped TiO_2_. Another reason is that Ta incorporation leads to a less dense film structure due to the donor doping induced oxygen vacancies defects to maintain charge neutral as commonly seen in doped metal oxides [16]. The relatively loose film structure of the Ta-doped TiO_2_ contributes to a higher film thickness.

**Figure 2:**
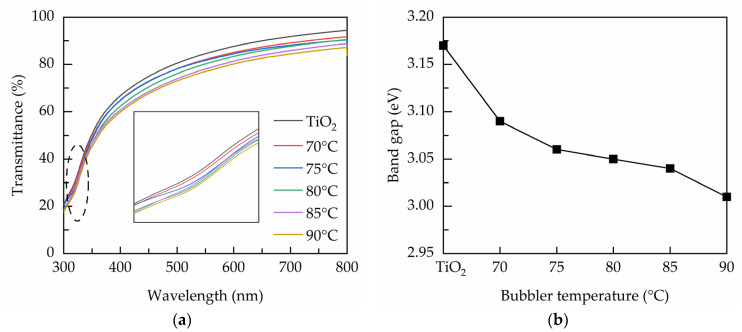
(**a**) Transmittance spectra and (**b**) band gap of the PEALD films prepared with various bubbler temperatures.

Figure 2a shows the transmittance over a wavelength range from 300 to 800 nm for the PEALD films prepared at various bubbler temperatures. The lower transmittance at the short wavelength region is related to the band-to-band absorption of the TiO_2_ films. The transmittance decreases with increasing the bubbler temperature, indicating that the increase of Ta concentration enhances the absorption. The reduced transmittance could also be a consequence of the light scattering caused by the oxygen vacancies [29]. The inset shows the transmittance in the short-wavelength region (300–400 nm) to evaluate the absorption edge. It can be seen that the edge shifts towards longer wavelength direction when the bubbler temperature increases, inferring the reduced band gap. Figure 2b shows the band gap deduced from Tauc’s equation [30]:

(3) $${\left(\alpha \mathrm{hv}\right)}^{n}=A\left(\mathrm{hv}-E_{g}\right)$$

where α is the absorption coefficient, hv is the photon energy, A is the material-dependent constant, and E_g_ is the band gap. The n value is taken as 1/2 for indirect band gap anatase TiO_2_ [31]. The band gap values of the PEALD films are evaluated by plotting (αhv)^1/2^ versus hv and extrapolating the linear region of the resultant curves to obtain an interception with the hv-axis. The band gap of the TiO_2_ is 3.17 eV, and it decreases from 3.09 to 3 eV by increasing the bubbler temperature from 70 to 90 °C. This is possibly related to the band gap narrowing effect due to the formation of the oxygen vacancies defects that introduce shallow donor levels below the conduction band. Similar effect can also be observed elsewhere [32].

**Figure 3:**
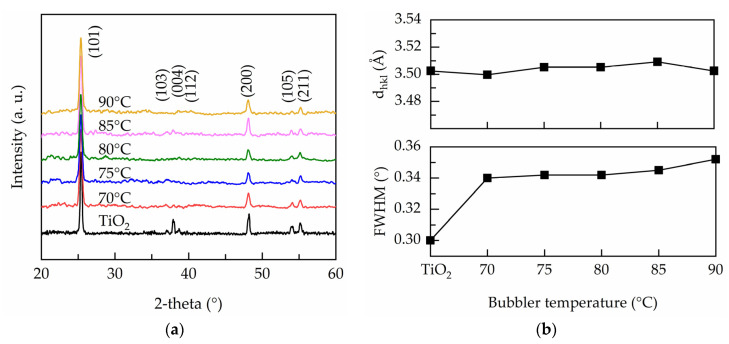
(**a**) XRD pattern, (**b**) interplanar distance and FWHM of the PEALD Ta-doped TiO_2_ films prepared at various bubbler temperatures.

Figure 3a shows the XRD patterns of the PEALD undoped and Ta-doped TiO_2_ films. The peaks labeled are well matched to anatase TiO_2_ (JCPSD#83-2243). With Ta doping, the (103), (004), and (112) peaks vanish, but no additional peak such as Ta or Ta_2_O_5_ is found, indicating that Ta atoms are well-incorporated into the TiO_2_ crystalline structure by replacing Ti^4+^ at random lattice sites. The full width at half maximum (FWHM) of the most intense (101) orientation is extracted to calculate crystallite size according to the Scherrer equation:

(4) $$L=\frac{K\lambda }{\beta cos\theta }$$

with *L* the crystallite size, *K* the shape factor taken as 0.9, *λ* the X-ray wavelength (0.154 nm), *β* is the width of the observed diffraction line at its half intensity maximum, and *θ* is the Bragg angle. Figure 3b illustrates the FWHM and crystallite size as function of bubbler temperature. It is seen that the FWHM increases when Ta is substituted into TiO_2_, suggesting that the crystallinity is diminished. The crystallite size reduces from 28 nm for undoped TiO_2_ to 23 nm for the film deposited at the bubbler temperature of 70 °C. Further increasing the bubbler temperature up to 90 °C results in a slight reduction in crystallite size. Although Ta^5+^ has a similar ionic radius to Ti^4+^, the electron density around Ta and the doping induced oxygen vacancies cause the rearrangement of the nearby atoms [16,33], which limits crystal growth.

**Figure 4:**
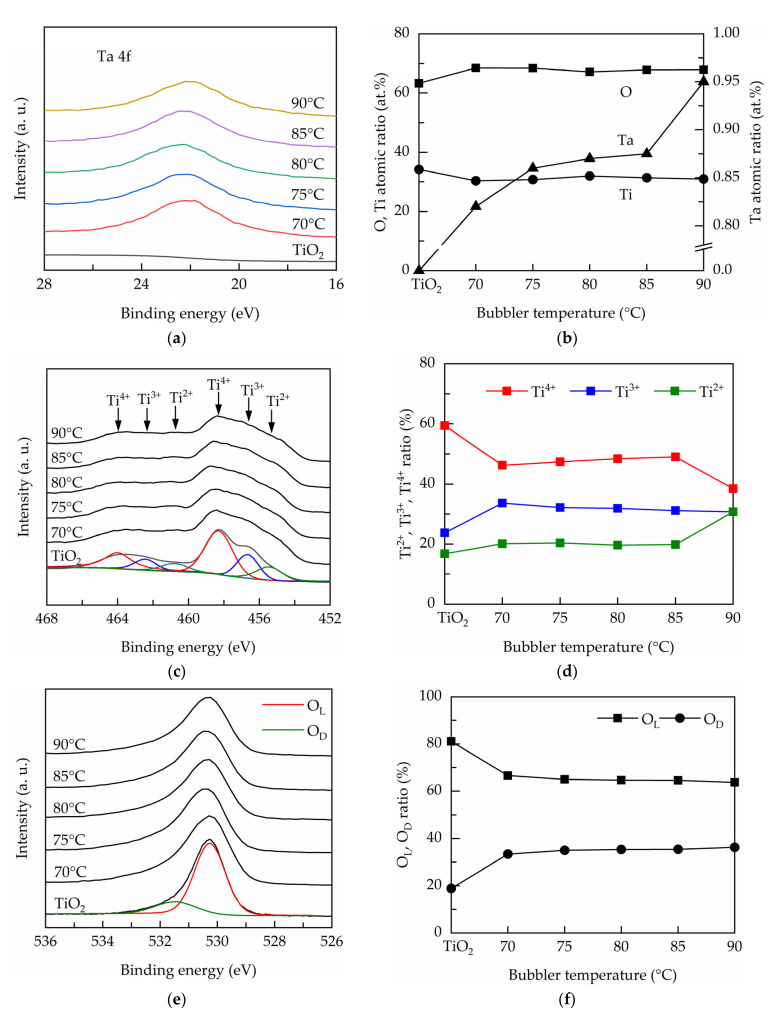
(**a**) Ta 4f spectra, (**b**) O, Ti and Ta atomic ratios, (**c**) O 1s spectra, (**d**) O_L_ and O_D_ peak area ratios, (**e**) Ti 2p_3/2_ spectra, and (**f**) Ti^2+^, Ti^3+^ and Ti^4+^ peak area ratios for the PEALD Ta-doped TiO_2_ films.

Figure 4 shows the composition and chemical states measured by XPS for the PEALD Ta-doped TiO_2_ films prepared at different bubbler temperatures. Figure 4a shows the high-resolution Ta 4f for the PEALD films, evidencing the Ta incorporation into the TiO_2_ films. The Ta peaks can be deconvoluted into two Gaussian components at 21.7 and 23.7 eV, corresponding to TaO_x_ (x < 2.5) [34].The atomic ratios of the Ti, O, and Ta elements are depicted in Figure 4b. The TiO_2_ film has an oxygen-deficient structure. The Ta content is varied from 0.82 to 0.95 at.% with increase in bubbler temperature from 70 to 90 °C. The XPS spectra over the biding energy of 450 to 470 eV corresponding to Ti 2p are shown in Figure 4c. The binding energies of around 453–460 eV and 460–466 eV belong to Ti 2p_3/2_ and Ti 2p_1/2_, respectively [35]. Each spin state can further be deconvoluted into Ti^4+^, Ti^3+^ and Ti^2+^ peaks [36]. The Ti 2p_3/2_ peaks are analyzed, and the peak ratios of Ti^2+^, Ti^3+^ and Ti^4+^ to total are shown in Figure 4d. The TiO_2_ has the highest T^i4+^ peak ratio of about 60%. At the bubbler temperature of 70 °C, the Ti^4+^ ratio decreases and Ti^3+^ ratio increases, indicating that Ta dopants create oxygen vacancies and reduce Ti^4+^ to Ti^3+^. At the bubbler temperature of 90 °C, the Ti^4+^ ratio further reduces and the Ti^2+^ ratio increases. This result suggests that the higher Ta incorporation causes Ti^4+^ to reduce to not only Ti^3+^ but also Ti^2+^. Figure 4e shows the high-resolution O 1s spectra for the PEALD films. The peaks are further split into two peaks, one at the lower binding energies (530–531 eV) assigned to lattice oxygen and the other at the higher binding energies of 531–532 eV ascribed to oxygen ions in oxygen-deficient regions in the lattice [37]. The latter is usually deemed to reflect the presence of oxygen vacancies. The ratio of each component to total is calculated as shown in Figure 4f. The lattice oxygen shows an opposite trend to defective oxygen. The increased defective oxygen ratio confirms that the Ta doping leads to the generation of oxygen vacancies defects.

**Figure 5:**
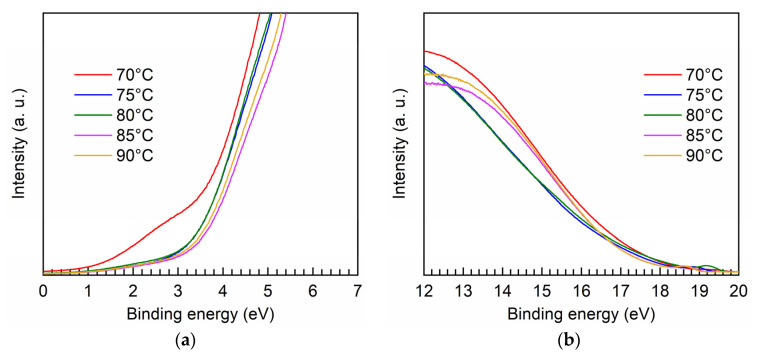
UPS spectra in the (**a**) low binding energy region and (**b**) high binding energy region for the PEALD Ta-doped TiO_2_ films prepared at different bubbler temperatures.

Figure 5 shows the UPS spectra for the PEALD Ta-doped TiO_2_ films prepared at different bubbler temperatures. As shown in Figure 5a, the extrapolation of the linear region of the curves to the binding energy axis gives the maximum valance band energy (E_VBM_) with respective to the Fermi energy. The curves in the high binding energy region are shown in Figure 5b, and similarly, the extrapolation of the linear region of the curves to the x-axis corresponds to the cut-off energy (E_cutoff_). Accordingly, the positions of Fermi level (E_F_) and valance band (E_v_) can be calculated as given by [38]:

(5) $$E_{F}=E_{\mathrm{cutoff}}-21.2,$$

(6) $$E_{v}=E_{F}-{\;E}_{\mathrm{VBM}}$$

where the value of 21.2 eV corresponds to the energy of the used UV light source. The position of the conduction band (E_c_) is further obtained using: E_c_ = E_v_ + E_g_ with the band gap values shown previously. The calculated E_c_ and E_v_ are summarized in Table 2. It is found the difference between the doped films is relatively small; however, the Ta-doped TiO_2_ films have both deeper conduction band and valence band edges, as compared to the undoped TiO_2_ film. The deep valance band positions favor the applications requiring hole blocking ability such as the electron transport layer in solar cells [39,40]. The lowered conduction band edges of the Ta-doped films indicate that Ta incorporation can adjust the conduction band position, which is benefit for interfacial band alignment engineering and a very important feature for being used as a selective charge transport layer of optoelectronics.

**Figure 6:**
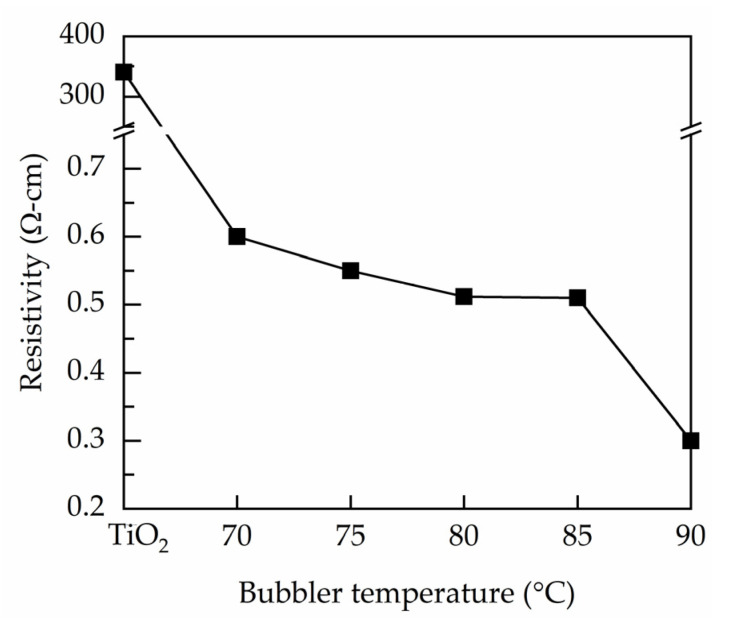
Four-point probe resistivity of the PEALD Ta-doped TiO_2_ films prepared at different bubbler temperature.

Figure 6 shows the resistivity measured by a four-point probe for the PEALD Ta-doped TiO_2_ films prepared at different bubbler temperatures. The TiO_2_ exhibits a considerably high resistivity of 3.4 × 10^2^ Ω·cm. The Ta incorporation leads to significant reduction in resistivity. All the Ta-doped TiO_2_ films have resistivities at the order of 10^−1^ Ω·cm. The minimum resistivity is 3 × 10^−1^ Ω·cm at the bubbler temperature of 90 °C. The decrease in resistivity is due to the Ta substitution for Ti atoms in the lattice sites, contributing free electrons. In addition, the Ta incorporation also results in creation of oxygen vacancies (as suggested by XPS), which are widely known as one of the sources of free electrons. Similar resistivity reduction due to the Ta doping is reported by other studies. For example, the resistivity of the Ta-doped TiO_2_ films prepared using metal organic chemical vapor deposition decreases four to six orders for at a high Ta doping level of 4–5% [17,41]. In the present work, the Ta content for the bubbler temperatures of 70–90 °C is between 0.82 and 0.95 at.%, leading to resistivities three orders lower than that of the undoped TiO_2_ film.

**Figure 7:**
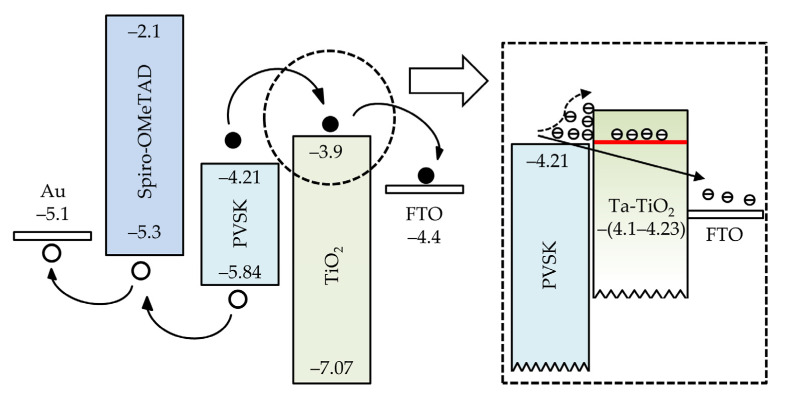
Isolated energy band diagram of each material involved in a TiO_2_ ETL-based PSC. The inset indicates the interfaces of perovskite/TiO_2_ and perovskite/Ta-doped TiO_2_ ETL.

Figure 7 depicts the isolated energy band diagram of conventional TiO_2_ ETL-based PSCs. The E_c_ and E_v_ values of each layer are experimentally determined. Similar values can be found in other studies [42,43,44,45]. The photogenerated electrons and holes move towards different directions. A downward shift of CBM is generally favorable in the viewpoint of electron transport. However, as shown in the inset of Figure 7, the undoped TiO_2_ layer has a shallower CBM than the perovskite absorber, which causes the band mismatch and obstacles for electron transport. The charge accumulation at the perovskite/TiO_2_ interface could resist electrons to flow into FTO. In the case of the adoption of Ta-doped TiO_2_, with the experimental E_c_ values around −(4.1–4.23) eV shown in the UPS result, the band mismatch is supposed to be mitigated or absent due to the deeper conduction band edge. Considering the conduction band of the perovskite layer (−4.21 eV), the Ta-doped TiO_2_ prepared at the bubbler temperature of 85 °C is used for device fabrication due to the least conduction band offset.

**Figure 8:**
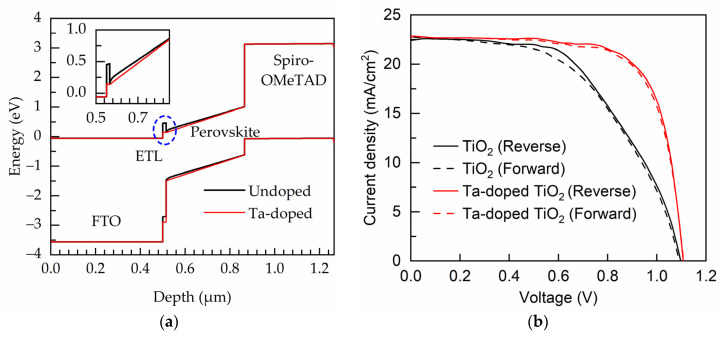
(**a**) Band bending of energy levels in PSCs. (**b**) J–V curves of the PSCs measured in reverse and forward directions. The inset shows the enlarged view at the ETL/perovskite interface.

Figure 8a shows the band-bending of energy levels in the PSCs with undoped TiO_2_ and Ta-doped TiO_2_ ETL deposited at the bubbler temperature of 85 °C. The major difference is at the ETL/perovskite interface. A conduction band barrier is clearly presented at the undoped TiO_2_/perovskite absorber interface; however, the barrier disappears in the case of using Ta-doped TiO_2_ ETL due to the very little conduction band mismatch. This is expected to be reflected on the improvement in FF of the devices. In addition, as shown in the inset, the slope of the conduction band of the Ta-doped TiO_2_ device is steeper than that of undoped TiO_2_ device, and this implies a greater built-in electric field, which could enhance the separation of photo-generated electron-hole pairs. The J–V characteristics of the fabricated PSCs in the forward and reverse scans are shown in Figure 8b, and the corresponded external photovoltaic parameters are listed in Table 3. Apart from the slightly improved V_oc_ and J_sc_, the cell with the Ta-doped TiO_2_ ETL has a considerably enhanced FF. The SEM images of the PEALD TiO_2_ and Ta-doped TiO_2_ films are shown in Figure S1. The two samples are compact without observable pinholes, and do not show much difference in surface morphology likely due to the low-Ta doping level. It is believed that the FF improvement of the perovskite solar cells with the Ta-doped TiO_2_ ETL is due to the reduction of the conduction band mismatch, thereby decreasing the transport barrier of electrons. This demonstrates the significance of the conduction band alignment of the ETL. In addition to the absence of the transport barrier at perovskite/ETL interface, the improved FF of the device with the Ta-doped TiO_2_ ETL is partially owing to the smaller series resistance as assessed from the inverse slope of the J–V curves at the V_oc_-point. The hysteresis index of the PSCs is investigated, which is given as [46]:

(7) $$\mathrm{Hysteresis}=\frac{\int_{\mathrm{OC}}^{\mathrm{SC}}J_{R}\left(V\right)\varphi \left(V\right)\mathrm{dV}-\int_{\mathrm{SC}}^{\mathrm{OC}}J_{F}\left(V\right)\varphi \left(V\right)\mathrm{dV}}{\int_{\mathrm{OC}}^{\mathrm{SC}}J_{R}\left(V\right)\varphi \left(V\right)\mathrm{dV}+\int_{\mathrm{SC}}^{\mathrm{OC}}J_{F}\left(V\right)\varphi \left(V\right)\mathrm{dV}},$$

where the subscript R represents reverse scan, F denotes the forward scan, and H is the unit step function. The result reveals the hysteresis of 1.06% for the device with undoped TiO_2_ ETL and 0.21% for the cell with Ta-doped TiO_2_ ETL. This is in agreement with the findings reported by Kim et al. [47], where PSCs with doped metal oxide ETLs exhibit less hysteresis compared to undoped metal oxides. It is worth mentioning that the Ta-doped TiO_2_ film prepared at the bubbler temperature of 90 °C has been also applied to PSC fabrication as it seems to have the lowest resistivity as well as an appropriate conduction band position. However, the resultant device conversion efficiency is lower than in the case of 85 °C-bubbler temperature. The J–V curve and the corresponded photovoltaic performance of the device at the bubbler temperature of 90 °C are shown in Figure S2. The device has conversion efficiency of 17.3% (V_oc_ of 1.1 V, J_sc_ of 22.5 mA/cm^2^, FF of 0.7), lower than that (18.09%) of the device with the Ta-doped TiO_2_ ETL deposited at 85°C. It is speculated that the performance reduction might be related to the increased defect formation when the impurity doping level increases. This compromises the cell performance, and thus the optimal Ta bubbler temperature occurs at 85 °C.

**Figure 9:**
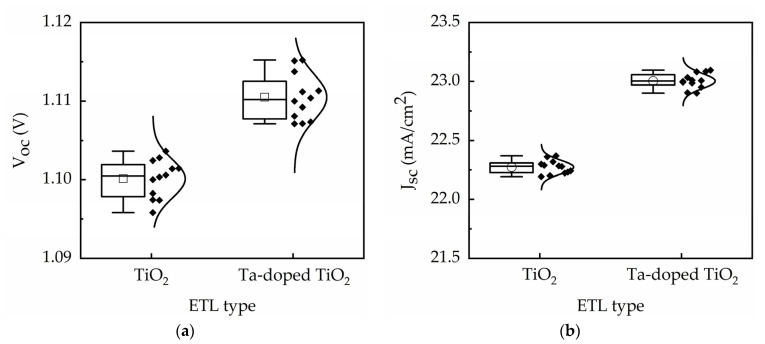

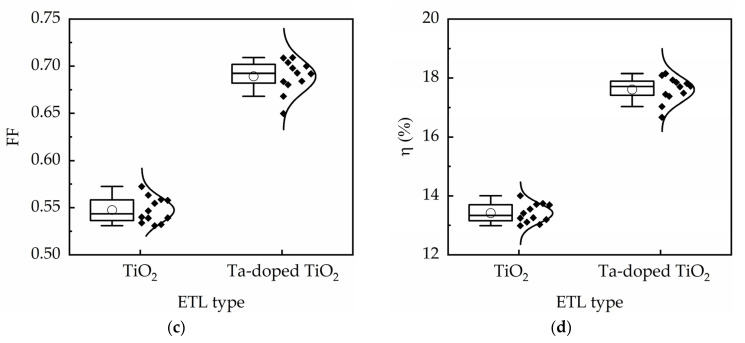
Performance of 12 devices for TiO_2_ and Ta-doped TiO_2_ perovskite solar cells: (**a**) V_oc_, (**b**) J_sc_, (**c**) FF, and (**d**) η.

The performance of 12 devices under reverse scan is shown in Figure 9 for TiO_2_ and Ta-doped TiO_2_ perovskite solar cells. The results demonstrate small standard deviations of the device photovoltaic parameters. For instance, the standard deviation of the device efficiency is less than 0.4% for TiO_2_ devices and 0.46% for Ta-doped TiO_2_ devices, indicating high reproducibility. Moreover, the average conversion efficiency has the same trend shown in Figure 9, inferring that the comparison of efficiency among different groups is reliable.

## 4. Conclusions

The Ta-doped TiO_2_ thin films are prepared using PEALD with co-injected Ta(OEt)_5_ and TTIP metal precursors as Ta and Ti sources, respectively. The bubbler temperature varies to obtain different Ta content in the films. The XRD result shows that the Ta incorporation does not lead to additional crystalline phases but reduces crystallinity. As confirmed by XPS, the Ta dopants cause Ti^4+^ ions reducing to Ti^3+^ and Ti^2+^, due to the creation of the oxygen vacancies defects. The resistivity significantly reduces by three orders at the Ta content of 0.82–0.95 at.%, as compared to that of the undoped TiO_2_. The band gap, conduction band, and valence band position can be tailored by the Ta incorporation, and this is favorable for the applications involving interfacial band structure engineering. Finally, the PSC with Ta-doped TiO_2_ ETL demonstrates a significantly improved FF from 0.54 to 0.71 and conversion efficiency from 13.42% to 18.09% as compared to the PSC with conventionally undoped TiO_2_ ETL.

## Figures and Tables

**Table 1:** Deposition parameters of the PEALD Ta-doped TiO_2_ films.

Parameter	Value
Substrate temperature (°C)	250
Bubbler temperature (°C)	70–90
Nitrogen carrier flow rate (sccm)	120
Metal precursor pulse time (s)	1.6
Metal precursor purge time (s)	6
O_2_ flow rate (sccm)	150
O_2_ pulse time (s)	11
O_2_ purge time (s)	5
O_2_ plasma power (W)	1500
Ar flow rate (sccm)	80
Post annealing temperature (°C)	500

**Table 2:** Band gap, valance band position, and conduction band position for the PEALD Ta-doped TiO_2_ films with different bubbler temperatures.

Bubbler Temperature (°C)	Band Gap (eV)	E_v_ (eV)	E_c_ (eV)
TiO_2_	3.17	−7.07	−3.9
70	3.09	−7.19	−4.1
75	3.06	−7.19	−4.13
80	3.05	−7.22	−4.17
85	3.04	−7.24	−4.2
90	3	−7.24	−4.23

**Table 3:** Performance of PSCs measured in the reverse and forward directions.

Parameter	J_sc_ (mA/cm^2^)	V_oc_ (V)	FF	η (%)
Undoped TiO_2_ (Forward)	22.9	1.09	0.55	13.02
Undoped TiO_2_ (Reverse)	22.3	1.10	0.54	13.42
Ta-doped TiO_2_ (Forward)	22.8	1.11	0.70	17.91
Ta-doped TiO_2_ (Reverse)	23.0	1.11	0.71	18.09

## Data Availability

Data sharing is not applicable to this article as no new data were created or analyzed in this study.

## Supplementary Materials

The following are available online at https://www.mdpi.com/article/10.3390/nano11061504/s1, Figure S1: SEM images of PEALD (a) TiO_2_ and (b) Ta-doped TiO_2_ deposited at the bubbler temperature of 85 °C, Figure S2: J–V curve of the perovskite solar cell with the Ta-doped TiO_2_ ETL deposited at the bubbler temperature of 90 °C.
