# Supplementary material for: Tantalum-Doped TiO2 Prepared by Atomic Layer Deposition and Its Application in Perovskite Solar Cells
Source: Nanomaterials (Basel). 2021 Jun 7;11(6):1504. doi: 10.3390/nano11061504 (PMC8226548; doi:10.3390/nano11061504)
Supplement: Supplementary file 1 [file nanomaterials-11-01504-s001.zip › nanomaterials-1223987-supplementary.pdf]

Supplementary Materials

Tantalum-Doped TiO_2 Prepared by Atomic Layer Deposition and its Application in Perovskite Solar Cells

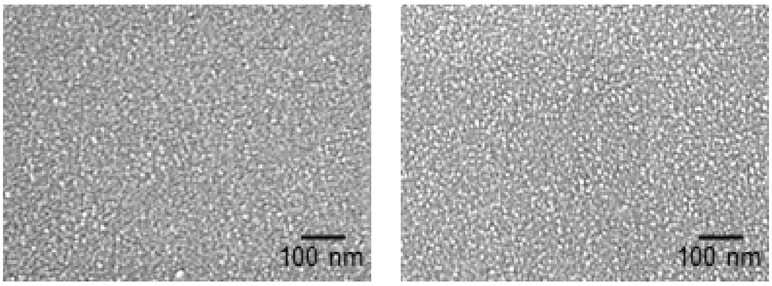

(a) (b)

Figure S1. SEM images of PEALD (**a**) TiO_2 and (**b**) Ta-doped TiO_2 deposited at the bubbler temperature of 85 °C.

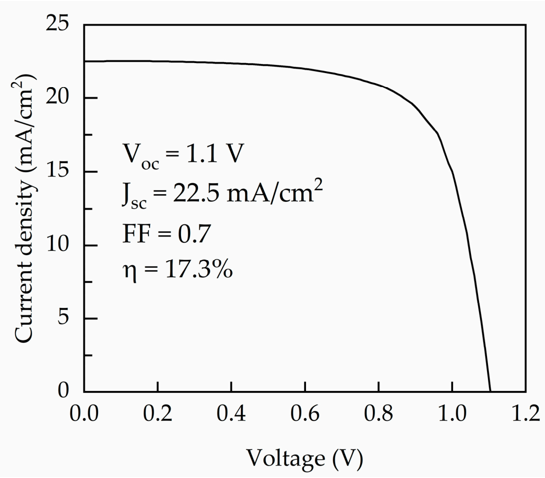

Figure S2. J–V curve of the perovskite solar cell with the Ta-doped TiO_2 ETL deposited at the bubbler temperature of 90 °C.
